# Effects of personality traits on the manifestations of irritable bowel syndrome

**DOI:** 10.1186/1751-0759-6-20

**Published:** 2012-10-30

**Authors:** Jun Tayama, Naoki Nakaya, Toyohiro Hamaguchi, Tadaaki Tomiie, Masae Shinozaki, Tatsuo Saigo, Susumu Shirabe, Shin Fukudo

**Affiliations:** 1 Center for Health and Community Medicine, Nagasaki University, Nagasaki, Japan; 2 Department of Preventive Medicine and Epidemiology, Tohoku Medical Megabank Organization, Tohoku University, Tohoku, Japan; 3 Department of Occupational Therapy, School of Health and Social Services, Saitama Prefectural University, Saitama, Japan; 4 School of Psychological Science, Health Sciences University of Hokkaido, Hokkaido, Japan; 5 Department of Behavioral Medicine, Tohoku University Graduate School of Medicine, Tohoku, Japan

**Keywords:** Personality, Irritable bowel syndrome, Depression, Neuroticism, Brain- gut interactions

## Abstract

**Objective:**

Previous studies have reported that patients with irritable bowel syndrome (IBS) show high neuroticism. However, the precise association between the IBS subtypes and the degree of neuroticism in younger populations is largely unknown. We tested our hypothesis that subjects with diarrhea-predominant IBS may have a higher degree of neuroticism than subjects without IBS or those with other subtypes of IBS. We also verified the additional hypothesis that the severity of neuroticism might be correlated with the severity of IBS in younger populations.

**Methods:**

We conducted a cross-sectional survey of 557 university students, ranging in age from 18 to 21 years. Presence/ absence of IBS and determination of the IBS subtype was by the Rome II Modular Questionnaire, while the severity of IBS was determined by the IBS severity index (IBS-SI). The degree of neuroticism was evaluated using the Maudsely Personality Inventory (MPI). The presence/absence of psychological distress was measured with the K6 scale.

**Results:**

Neuroticism scores in the subjects with diarrhea-predominant IBS were significantly higher than those in the non-IBS subjects or subjects with constipation-predominant IBS. The neuroticism scores were significantly correlated with the IBS-SI scores in all subjects with IBS.

**Conclusion:**

These results suggest that neuroticism is involved in the pathophysiology of IBS in young subjects, especially in that of the diarrhea-predominant subtype.

## Introduction

Irritable bowel syndrome (IBS) is a functional gastrointestinal disorder not associated with major organic diseases [1,2]. The characteristic pathophysiological features of IBS are dysmotility of the lower gastrointestinal tract [3], visceral hypersensitivity [4], and psychological abnormalities [5]. A previous study focusing on the psychological abnormalities in IBS reported that the gastrointestinal symptoms of IBS worsened with aggravation of the psychological state and that they improved with recovery of the psychological state [5]. Several epidemiologic studies have revealed that the prevalence rates of depression and anxiety are higher in IBS patients than those in healthy persons [6-8]. In a study that examined the depressive symptoms in 80 IBS patients and 21 healthy persons aged 21 years to 65 years old, the average scores for depression were higher in the group with constipation-predominant IBS (C-IBS) and the IBS group with alternating diarrhea and constipation (A-IBS) than in the healthy persons; however, no significant difference in the score for depression was seen between the subjects with diarrhea-predominant IBS (D-IBS) and healthy persons [9].

Among the several known personality traits, neuroticism has been suggested as a risk factor for the development of IBS [10-13]. In the study conducted in 41 IBS patients and 2000 healthy persons aged 41 to 49 years old by Palmer et al. using [10] Eysenck’s index (Maudsely Personality Inventory: MPI), the average score for neuroticism was higher in the IBS patients than that in healthy persons. In a study performed in 60 IBS patients (average age ± standard deviation: 29 ± 7) and 55 healthy persons (average age ± standard deviation: 27 ± 7) using the Minnesota Multiphasic Personality Inventory (MMPI-2), Mousavinasab et al. [11] reported a higher average score for hypochondriasis in the IBS patients than that in the healthy persons. Farman et al. [12] showed that the average score for neuroticism in an evaluation conducted using the NEO Five-Factor Inventory (NEO-FFI) was significantly higher in the C-IBS group (33 patients) than the scores in the groups with other subtypes of IBS (71 with D-IBS patients and 46 A-IBS patients). In the study conducted by Tanum et al. [14] in 56 patients with functional gastrointestinal disorders (FGIDs), including 31 IBS patients and 55 age-matched healthy persons (age range: 18–70 years old), the average score for neuroticism on the NEO Personality Inventory (NEO-PI) was higher in the FGID patients than that in the healthy persons. Thus, neuroticism may indeed play some role in the pathophysiology of IBS.

In an epidemiologic survey of 10,000 Japanese adults aged over 20 years old, the prevalence rate of IBS in persons aged 20–29 years, as diagnosed based on the ROME-III criteria, was 14% among males and 22% among females [15]. In persons aged 30 years or over, the prevalence rate of IBS decreased gradually with increasing age [15]. In an investigation of 1,087 Japanese college students with an average age of 20 years (SD ± 2), the prevalence rate of IBS, as diagnosed based on the ROME-III criteria, was 19% overall, 17% in males, and 20% in females [16]. The above reports suggest that the prevalence of IBS may be higher in college students as compared with that in older age groups, which warrants further analysis.

Despite the earlier studies mentioned above, there is no evidence yet to show that neuroticism is a risk factor for the onset of any subtype of IBS in young patients. Also, no study has been performed yet to determine the relationship between the severity of neuroticism and the severity of IBS among subjects with IBS. Investigation of the relationship between the severity of neuroticism and the severity of IBS in subjects with each subtype of IBS may clarify the physiological relationship between neuroticism and the development of gastrointestinal symptoms. Neuroticism has been linked to the development of mood and anxiety disorders [17,18]. Anxiety has been shown to play a role in patients with diarrhea-predominant IBS [19]. Therefore, neuroticism may be closely related to the development of D-IBS. In this research, we attempted to verify the following two hypotheses in university students, who represent the young population.

1) In the young population, subjects with diarrhea-predominant IBS show a higher degree of neuroticism than subjects not suffering from IBS or subjects with other IBS subtypes.

2) In the young population, the neuroticism score is correlated with the severity of IBS.

## Method

### Subjects

**Figure 1:**
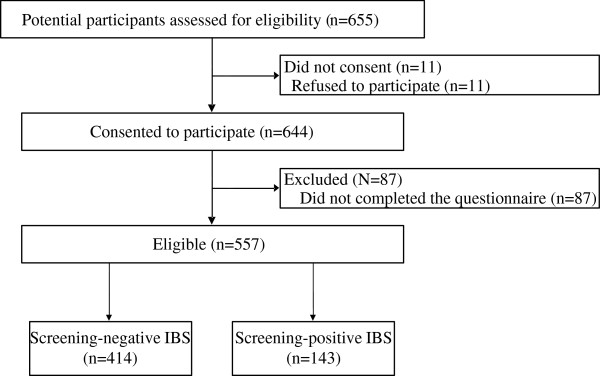
**Study flow.** Of 655 subjects, 11 did not provide consent for participation, and 87 did not respond to the questionnaire. We analyzed the data of the remaining 557 persons who answered all the items of the questionnaire.

We conducted a cross-sectional study of 655 university students, ranging in age from 18 years to 21 years. Of the 655 subjects, 11 refused to provide consent for the use of their data for research/participation in this study, and 87 did not respond to the questionnaire. We therefore analyzed the data of the remaining 557 persons who answered all the items of the questionnaire (Figure 1). Of these 557 subjects, 143 (26%) were diagnosed as having IBS based on responses to the IBS screening questionnaire described below.

### Measurements

#### The Rome II Modular Questionnaire (RIIMQ)

Rome II criteria, described below, are widely used for the diagnosis of IBS [20]: at least 12 weeks, which need not be consecutive, during the preceding 12 months during which the patient suffered from abdominal discomfort or pain satisfying the following three features: (1) relieved by defecation, (2) onset associated with a change in the stool frequency, and/or (3) onset associated with a change in the form (appearance) of the stool. The following symptoms cumulatively support the diagnosis and subtyping of IBS: (a) fewer than 3 bowel movements a week; (b) more than 3 bowel movements a day; (c) hard or lumpy stools; (d) loose (mushy) or watery stools; (e) straining during a bowel movement; (f) urgency (having to rush to have a bowel movement); (g) feeling of incomplete evacuation; (h) passage of mucus; (i) bloating or feeling of abdominal distension. D-IBS is defined by one or more of b, d or f and none of a, c or e, or 2 or more of b, d, or f and one of a or e. C-IBS is defined by one or more of a, c, or e and none of b, d or f, or 2 or more of a, c, or e and one of b, d or f. IBS patients not fulfilling the aforementioned criteria for D-IBS or C-IBS were defined as having A-IBS in this study. RIIMQ has been reported as a reliable tool for epidemiological surveys [21]. The Japanese version of the RIIMQ is a questionnaire that is used for both diagnosis of IBS and for determination of the subtype of IBS [22].

#### IBS severity

The Japanese version [22] of the IBS severity index (IBS-SI) [23] was used in this study to assess the severity of the lower gastrointestinal symptoms and the degree of impairment of the quality of life by the IBS. This instrument is composed of five items, with the possible total score in the range of 0 to 500. The IBS-SI scores the severity of the abdominal pain, abdominal distension, bowel movements, and the quality of life.

#### Psychological distress

The six-item K6 [24] was used in this study to evaluate the presence/absence of psychological distress in the subjects. The K6 has been developed to identify persons who are at a risk of developing mental states such as depression and anxiety [25]. The K6 total score can range from 0 to 24, and patients with scores of 13 or greater are categorized as suffering from psychological distress.

#### Personality

The Maudsely Personality Inventory (MPI) [26] is a measure used to assess personality in two dimensions, namely, neuroticism and extroversion/introversion. It is composed of 80 items calling for a yes/no response; Extroversion (E): 24-items; Neuroticism (N): 24-items; Lie (L): 20-items; Filler items: 12-items.

### Procedure

The questionnaire survey was given during an academic session in the classroom. First, we explained the research content and provided both a written and verbal explanation about the intended use of the data. In addition, we explained that persons not providing consent for participation in the study would not be placed at any disadvantage whatsoever. We only included the data of those students who agreed to cooperate with the research for the analysis in this study.

### Data analysis

Data are presented as means ± standard deviation (SD). An unpaired *t*-test was used to compare any two groups. Correlation coefficients were calculated by determination of the Pearson’s correlation coefficients. Chi-square test was used to evaluate differences in percentages. One-way ANOVA was performed to compare data between groups. One-way ANOVA was performed for between group comparisons. SPSS, version 18.0, was used for the statistical analyses.

### Ethics

The study protocol was approved by the Ethics Committee of Nagasaki University, and informed consent was obtained from all the subjects.

## Results

The demographic data of the study subjects and the reference values in healthy persons established in a previous study are shown in Table 1[16,27-31]. Males comprised 59% of all subjects (95% confidence interval (CI), 55 to 63%). The mean (±SD) age of the subjects was 19 (±1) years. The prevalences of D-IBS, C-IBS and A-IBS in the study population were 4% (95% CI, 3 to 6), 9% (95% CI, 7 to 12) and 12% (95% CI, 10 to 15), respectively. In addition, the prevalence of psychological distress in the overall study population was 7% (95% CI, 5 to 9), consistent with the finding of a previous study [30].

**Table 1:** Demographic data and reference values

**Variables**	**All subjects (n = 557) (95% CI)**	**Reference value (general population (95% CI))**	
Sex (male (%))	59 (55–63)	47 (36–45)	Sugaya and Nomura[16]
Age	19 ± 1 (19–19)	-	-
IBS^+^ (%)^a^	26 (22–30)	14 (12–15)	Kubo M, et al. [27]
D-IBS (%)^b^	4 (3–6)	4 (−)	Kubo M, et al. [27]
C-IBS (%)^c^	9 (7–12)	3 (−)	Kubo M, et al. [27]
A-IBS (%)^d^	12 (10–15)	3 (−)	Kubo M, et al. [27]
IBS-SI	34.1 ± 44.2 (30.4-37.8)	30 (−)^g^	Tana C, et al. [28]
K6^e,f^	5.4 ± 4.5 (5.0-5.7)	3.6 ± 3.9 (−)	Sakurai K, et al. [29]
Persons with psychological distress (%)^g^	7 (5–9)	7 (7–7)	Kuriyama S, et al. [30]
MPI	-	-	-
Extroversion	27.2 ± 10.3 (26.3-28.0)	27. 2 ± 7.0 (−)	Iwawaki, S et al. [31]
Neuroticism	21.6 ± 10.7 (20.7-22.5)	21.6 ± 7.9 (−)	Iwawaki, S et al. [31]
Lie score	13.4 ± 5.8 (12.9-13.8)	13.4 ± 5.1 (−)	Iwawaki, S et al. [31]

Data are expressed as mean ± standard deviation. CI, confidence interval.

^a-e,g^ The numerical values represent the percentage of the total number of participants, namely, IBS patients plus healthy persons.

^f^Persons with a score of ≥13 points (out of a total 24 points) on the K6 were defined as experiencing psychological distress.

The data (score) represent median values.

The scores for each of the evaluated items in the IBS group and non-IBS group are shown in Table 2. The proportion of males/females in the non-IBS group was 64%/36%, and that in the IBS group was 48%/52%, with a significant difference in the proportion of males/females between the two groups (p = 0.0001). Scores on the IBS-SI in the IBS group (53.9 ± 50.2) were significantly higher than those in the non-IBS group (27.3 ± 39.8, p = 0.0001). Scores on K6 in the IBS group (6.7 ± 5.0) were also significantly higher than those in the non-IBS group (4.9 ± 4.2, p = 0.0001). The differences in the scores on the IBS-SI and K6 were seen even after adjustment for the sex distribution (p = 0.0001 for all). The percentage of persons with psychological distress was significantly higher in the IBS group than that in the non-IBS group (11% vs. 5%, p = 0.0081).

**Table 2:** Difference between non-IBS group and IBS subgroups in the personality scales

**Variables**	**Non-IBS group (n = 414)**	**IBS group (n = 143)**	**IBS subgroup**			**p value**^**c**^
			**D-IBS**	**C-IBS**	**A-IBS**	
			**(n = 24)**	**(n = 50)**	**(n = 69)**	
Sex (male (%))	64	48 ^§^	58	32	55	p = 0.0001
Age	19 ± 1	19 ± 1	19 ± 1	19 ± 1	19 ± 1	p = 0.1479
IBS-SI	27.3 ± 39.8	53.9 ± 50.2 ^†^	47.9 ± 39.5 ^**^	51.6 ± 56.0 ^**^	57.6 ± 49.3 ^**^	p = 0.0001
K6^a^	4.9 ± 4.2	6.7 ± 5.0 ^†^	8.3 ± 5.1 ^**^	5.5 ± 4.0	7.1 ± 5.4 ^**^	p = 0.0001
Persons with psychological distress (%)^b^	5	11 ^§^	13	6	15	p = 0.0001
EPI	-	-	-	-	-	-
Extroversion	27.6 ± 10.2	25.8 ± 10.4	25.3 ± 10.8	27.1 ± 9.2	25.1 ± 11.2 ^*^	p = 0.2228
Neuroticism	21.4 ± 10.8	22.4 ± 10.4	26.0 ± 9.4 ^* ¶^	20.5 ± 11.5	22.5 ± 9.6	p = 0.1430
Lie score	13.3 ± 5.7	13.4 ± 6.0	12.4 ± 5.8	14.1 ± 6.6	13.3 ± 5.7	p = 0.6855

^a^Persons with scores of ≥13 points (out of a total 24 points) on the K6 were defined as experiencing psychological distress.

^b^Values are the percentages of the total number of participants (i.e., IBS group plus non-IBS group).

^c^One-way ANOVA was performed using the data from the non-IBS group, and the groups with the diarrhea-type, constipation-type and alternating-type IBS.

^†^p < 0.01 vs. non-IBS group; *p < 0.05 and **p < 0.01 vs. non-IBS group, unpaired *t*-test.

^§^p< 0.01 vs. non-IBS group, chi square test.

^¶^p < 0.05 vs. C-IBS group, unpaired *t*-test.

The scores for each of the evaluated items in the non-IBS group and the groups with each subtype of IBS are shown in Table 2. The main effects were seen in the scores on the IBS-SI (F (3,553) = 14.1, p = 0.0001) and K6 (F (3,553) = 3.6, p = 0.0001), as calculated by ANOVA. The percentage of persons with psychological distress was 13% in the D-IBS group, 6% in the C-IBS group, and 15% in the A-IBS group, and significant differences among the three groups were revealed by the chi-square test (*χ*^2^ = 10.5, p = 0.0146). Scores on the IBS-SI in all of the D-IBS (47.9 ± 39.5, p = 0.0109), C-IBS (51.6 ± 56.0, p = 0.0002) and A-IBS (57.6 ± 49.3, p = 0.0001) groups were significantly higher than the score in the non-IBS group (27.3 ± 39.8). Scores on K6 in the D-IBS group (8.3 ± 5.1, p = 0.0001) and the A-IBS group (7.1 ± 5.4, p = 0.0001) were significantly higher than the score in the non-IBS group (4.9 ± 4.2). Scores for extroversion as assessed by the MPI in the A-IBS group were significantly lower than those in the non-IBS group (25.1 ± 11.2 vs. 27.6 ± 10.2, p = 0.0305). Scores of neuroticism in the D-IBS group (26.0 ± 9.4) were significantly higher than those in the non-IBS group (21.4 ± 10.8, p = 0.0182). Furthermore, the scores for neuroticism in the D-IBS (26.0 ± 9.4) were significantly higher than those in the C-IBS group (20.5 ± 11.5, p = 0.0183). The differences in the scores on the IBS-SI and K6, and in the scores for extroversion, and neuroticism, between the two groups were seen even after adjustment for the sex distribution (p = 0.0001 for all).

**Figure 2:**
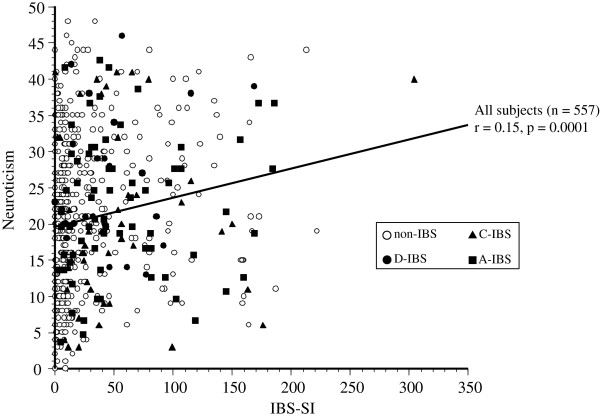
**Correlation between neuroticism and the total score of the IBS severity index in the study population.** A significant correlation was observed between the score for neuroticism and the IBS-SI score in all the subjects (r = 0.15, p = 0.0001). The corresponding correlation was r = 0.12 (p = 0.0001) in the C-IBS group and r = 0.09 (p = 0.0440) in the A-IBS group, both representing weak correlations. There was no significant correlation between the scores for extroversion and the IBS-SI scores.

**Figure 3:**
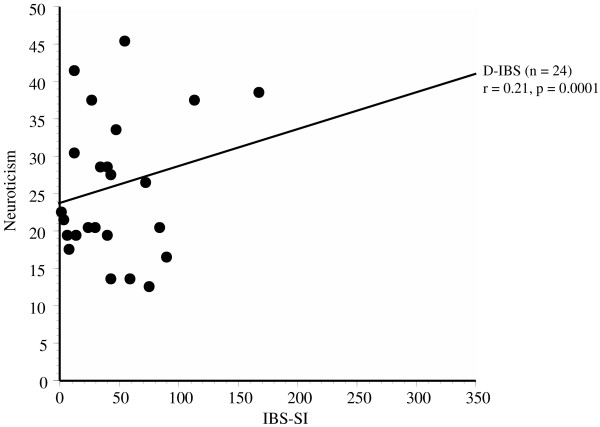
**Correlation between the score for neuroticism and the total score of the IBS-SI in the D-IBS subjects.** A significant correlation was observed between the scores for neuroticism and the IBS-SI scores in the D-IBS subjects (r = 0.21, p = 0.0001).

A significant correlation was observed between the scores for neuroticism and the IBS-SI scores in all the subjects (p = 0.0001), as shown in Figure 2. A significant correlation was observed between the degree of neuroticism and the IBS-SI score in the D-IBS patients (r = 0.21, p = 0.0001), as shown in Figure 3. The corresponding correlation was r = 0.12 (p < 0.01) in the C-IBS group, r = 0.09 (p < 0.05) in the A-IBS group, and r = 0.15 (p < 0.01) in the non-IBS group, both representing only weak correlations. There was no significant correlation between the scores for extroversion and the IBS-SI scores.

## Discussion

The results of our cross-sectional study performed in a young population of university freshmen revealed higher degrees of neuroticism in patients with D-IBS, diagnosed based on the ROME criteria, than in subjects not suffering from IBS and those diagnosed as having C-IBS. Therefore, hypothesis 1 was supported. Furthermore, the correlation between the degree of neuroticism and the severity of IBS also supported hypothesis 2.

A previous report indicated that among patients with IBS, the severity of neuroticism was higher in patients with C-IBS than that in patients with other subtypes of IBS [12]. However in this study, in contrast to the aforementioned report, the neuroticism score was higher only in the D-IBS group as compared with that in the non-IBS group. According to another previous study, while the scores for depressive mood were higher in the C-IBS and A-IBS groups than the score in healthy people, the scores did not differ significantly between the D-IBS group and healthy persons [9]. Furthermore, a survey of 1,087 Japanese college students revealed no differences in the average scores for depressive mood between patients with D-IBS or C-IBS and healthy persons [16]. Although Sugaya and Nomura reported that males comprised 64% of the D-IBS group but only 22% of the C-IBS group in their series [16], males in this study accounted for 58% and 32% of the patients in the D-IBS and C-IBS groups, respectively. It has been reported that, among Japanese adults, the prevalence of psychological distress, as judged by K6, is higher in females than in males [30], and that the prevalence rate of IBS is higher in females than in males [32]. Based on the higher percentage of females in the D-IBS group in this study, such a result may have been output in this study.

The following reason is speculated to explain the correlation between the neuroticism scores and the scores on IBS-SI in the D-IBS group in this study. Neuroticism in patients with depression has been reported to increase the score for depressive mood [33,34]. In patients with depression, the hypothalamic-pituitary-adrenocortical (HPA) axis is reported to be activated [35-37]. Therefore, the HPA-axis is assumed to also be activated in subjects with a high severity of neuroticism. In animal experiments, corticotropin-releasing hormone (CRH) has been reported to increase the fecal pellet output and exaggerate colonic motility [38]. A previous study reported HPA hyperactivity in adults with a high degree of neuroticism [39]. In IBS patients, exogenous CRH administration aggravates the brain-gut axis [40] and administration of CRH antagonists normalizes the axis [41]. Therefore, a high degree of neuroticism may aggravate the symptom of diarrhea by worsening the depressive mood and enhancing the activity of the HPA-axis, especially in younger age groups. Furthermore, in this study, subjects were categorized as having/not having IBS based on the results of a questionnaire, and not by a physician. Thus, the rate of IBS identified in this study may be an overestimate as compared with the potential diagnosis rate by a physician. If the prevalence rate of IBS found in this study were indeed higher than the potential prevalence rate determined based on a physician’s diagnosis, it is possible that our results may include the data of persons with relatively minor symptoms. Therefore, if the study were conducted only with patients with physician-diagnosed IBS, the relationship between psychological traits and the severity of IBS may have become more apparent, and the relationship may have been underestimated in this study.

In this study, no difference in the neuroticism score was observed between the subjects with and without IBS. By contrast, in some earlier studies, higher neuroticism scores have been reported in patients with IBS than in subjects without IBS [10,11]. A possible major reason for the absence of any significant difference in the neuroticism score between subjects with and without IBS in our study was that the subjects were all freshmen college students. The frequency of major depression has been reported to increase with age [42]. Neuroticism in patients with depression has been reported to raise the score for depressive mood [33,34]. Therefore, the lack of increased score for neuroticism in the IBS subjects overall, and of a correlation between the degree of neuroticism and the severity of IBS in the overall subject population in this study may suggest a subliminal role of neuroticism in IBS pathophysiology. Moreover, the hypothesis of involvement of neuroticism in the development of IBS may have originated from the relationship between the degree of neuroticism and that of the D-IBS phenotype in the younger generation.

The strength of this study is that it examined the involvement of neuroticism in college students with each subtype of IBS, and it was the first study to do so in college students. There are three limitations to this study. First, this research targeted only one university. The prevalence rates of IBS differ by culture [43,44]. The target university is located in a medium-sized city on the island of Kyushu in western Japan. Therefore, it is unknown whether the results can be extrapolated to Japanese university students in general. Second, since this study is a cross-sectional study, it could not be judged how the presence/degree of neuroticism influenced the severity of diarrhea in the subjects. Therefore, prospective studies are required. Third, although we evaluated psychological distress in the subjects using K6 in this study, we did not sufficiently evaluate the prevalence of depression. The lack of adequate evaluation of depression in the present study might limit the interpretation of the obtained results.

In conclusion, in a young study population in Japan, patients with D-IBS showed higher degrees of neuroticism as compared to subjects not suffering from IBS and subjects with C-IBS. Furthermore, the degree of neuroticism was correlated with the severity of IBS in the young subjects. This suggests that neuroticism may be involved in the onset and aggravation of IBS, and therapeutic approaches for D-IBS should take this finding into consideration.

## Abbreviations

IBS: Irritable bowel syndrome; C-IBS: Constipation-predominant IBS; D-IBS: Diarrhea-predominant IBS; A-IBS: IBS with alternating diarrhea and constipation; SD: Standard deviation; CI: Confidence interval.

## Competing interests

We have no conflict of interest to disclose.

## Authors’ contributions

JT designed and coordinated the study; NN, TH, TT, and MS provided the information about the psychological variables; TS supported the analysis of the data; MH, SS, and SF supported the interpretation of the data. All authors have read and approved the final manuscript.
